# Lung Function Decline in Pulmonary Alveolar Microlithiasis

**DOI:** 10.7759/cureus.74430

**Published:** 2024-11-25

**Authors:** Pier Valerio Mari, Angelo Coppola, Francesco Macagno

**Affiliations:** 1 Internal Medicine, Ospedale San Carlo di Nancy, Rome, ITA; 2 Pulmonary Medicine, San Filippo Neri Hospital, Rome, ITA; 3 Pulmonary Medicine, Fondazione Policlinico Universitario Agostino Gemelli IRCCS, Rome, ITA

**Keywords:** decline, progression, pulmonary alveolar microlithiasis, respiratory failure, spirometry

## Abstract

Pulmonary alveolar microlithiasis (PAM) is a rare lung disorder characterized by calcium phosphate microliths in the alveolar spaces. Autosomal recessive mutations on the SLC34A2 gene lead to altered type IIb sodium phosphate cotransporter in alveolar type-II cells of the lung, thus resulting in aggregations of microliths in the alveoli. To date, more than 1000 cases have been reviewed by expert pulmonary clinicians. PAM is observed worldwide, with numerous cases reported in Asia and Europe. It generally progresses slowly, with symptoms commonly emerging during an individual's third or fourth decade of life. Dissociation between the clinical picture and the radiological pattern is usual. Computed tomography (CT) may show extensive radiological disease even in patients with minimal clinical symptoms. The decline in lung function is typically progressive; however, detailed information regarding specific spirometry changes remains insufficient and largely unknown. PAM management is basically supportive using vaccines, antibiotics in recurrent infections, or long-term oxygen when respiratory failure is determined. A bilateral lung transplant may be a resolutive treatment for end-stage disease. We report a lung function decline of a familial case of PAM in a 71-year-old female patient at our Interstitial Lung Disease Clinic.

## Introduction

Pulmonary alveolar microlithiasis (PAM) is a rare genetic disorder included in metabolic lung diseases [1]. PAM is associated with autosomal recessive mutations [2] that affect the production of sodium-dependent phosphate cotransporter due to the inactivation of the SLC34A2 gene [3]. As a consequence, microliths can be developed from the intra-alveolar calcium and phosphate deposition by the alveolar type II cells. The majority of patients are asymptomatic at the time of PAM diagnosis even though the review of more than 1000 worldwide cases [4] points to a progressive decline that leads to fatal pulmonary failure. Long-term prognosis is primarily affected by the lack of effective treatments. The use of steroids or recurrent bronchoalveolar lavages did show a poor outcome, and only anecdotal reports suggested disodium etidronate as a potential therapeutic candidate [5]. End-stage cases may only benefit from bilateral lung transplantation, and recurrence of the disorder has never been described yet. To date, the management of patients affected by PAM is limited to the use of supplemental oxygen, treatment of comorbidities, low-trait respiratory infections, influenza vaccination, and palliative care.

## Case presentation

**Figure 1:**
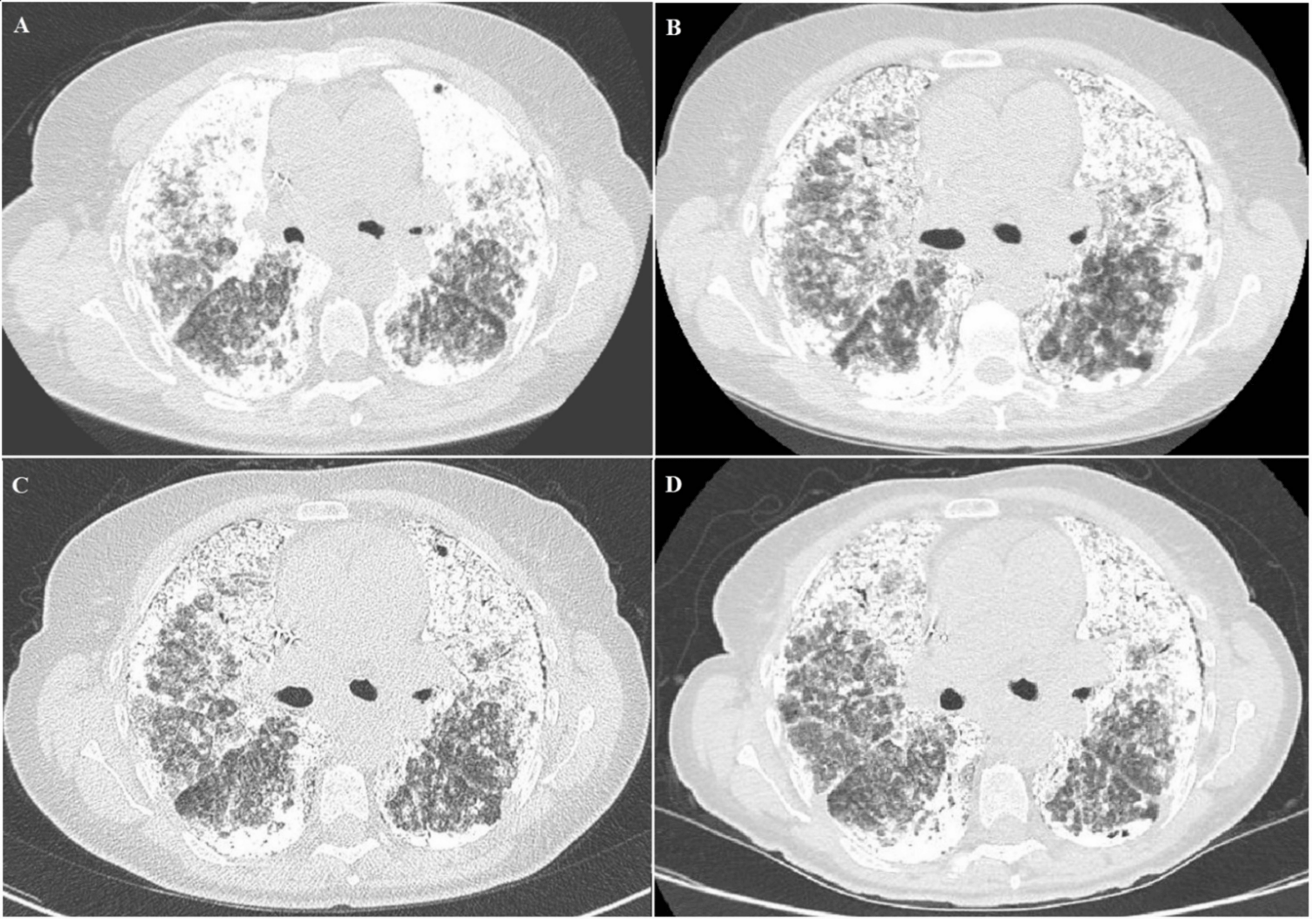
Radiological progression HRCTs: high-resolution CT scans High-resolution CT scans were performed in our institution during the long-term follow-up. HRCTs were performed at 22 years (A), 27 years (B), 28 years (C), and 31 years (D) since PAM diagnosis. No clear radiological progression was demonstrated during the last nine years of follow-up

We report the case of a 70-year-old female with PAM with frequent hospital admissions for pulmonary support and management of respiratory failure during the last three decades. This rare condition was accidentally diagnosed at the age of 39 with a chest X-ray because of the recent PAM diagnosis of her brother [6]. The first radiological study did show dense reticulonodular opacities in both lungs with a “sandstorm” appearance and a black pleura sign in the context of excellent clinical conditions despite a dry cough during the last few months. At the time she was diagnosed with PAM, her brother presented a rapid disease progression toward respiratory failure and was enlisted for a bilateral lung transplant. He died from infectious complications in a few months after the transplant. Moreover, since the first years of diagnosis, our patient has shown a worsening of the clinical picture, and we solicited the enrollment in transplant. Nevertheless, she adamantly refused because of her brother’s experience. Thus, she was referred to our institution for management and long-term follow-up. A series of high-resolution omputed tomography (CT) scans were performed over the years (Figure 1) and evidenced copious bilateral amounts of centrilobular dense opacities in association with an interstitial interlobular thickening. The upper and lower lobes were mostly affected, and the radiological follow-up suggested a stable presence of the small alveolar densities without an apparent evolution during the time. We performed repeated bronchoalveolar lavages in an attempt to reduce the calcified microliths in the alveolar lumen, but no clinical benefit was observed. During the last three decades, we attempted a few trials with high-dose steroids (1 mg/kg) without definitive improvements, except a small relief of the dyspnea on exertion. Since the diagnosis, the patient was scheduled for regular follow-up evaluations with lung function tests.

**Figure 2:**
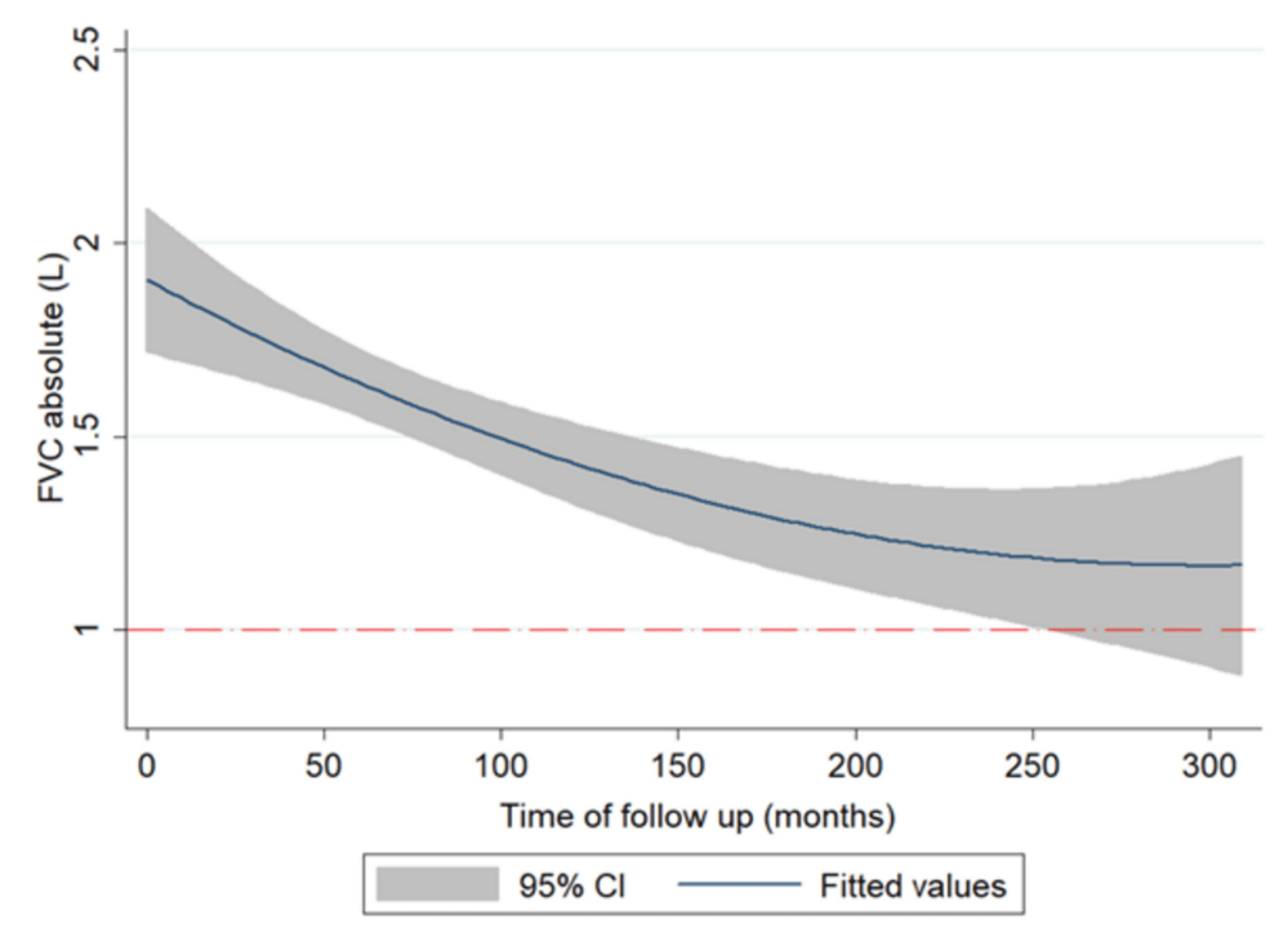
Spirometry changes Spirometry changes of absolute value of the forced vital capacity (FVC) during the follow-up

**Figure 3:**
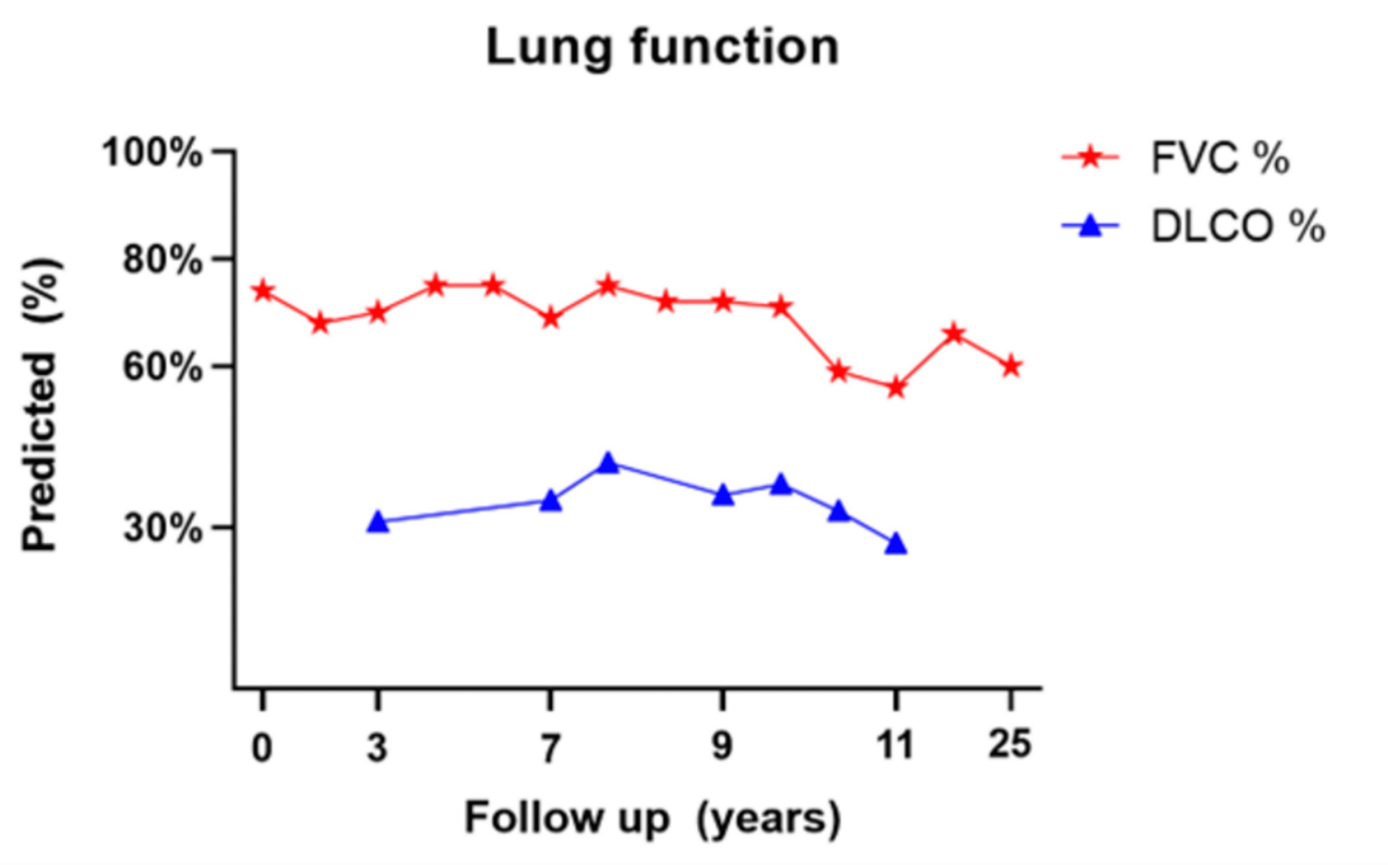
Spirometry changes of FVC and DLCO Spirometry changes of forced vital capacity (FVC) % predicted and diffusion in the lung of carbon oxide (DLCO) % predicted during follow-up

In Figure 2, we display the change in spirometry results during the last 25 years of follow-up: last tests have shown a generally steady decline of the forced vital capacity (FVC) when compared to the worse deterioration of the first 10 years. The analysis of the diffusion of carbon oxide (DLCO) progression was limited to a part of the full-length follow-up. Nevertheless, it exhibited a stable and low DLCO predicted value along with the appointments (Figure 3).

The analysis of the rate of decline of FEV1% during the 25 years of follow-up is remarkable (Figure ) and shows a rapid lung function decline during the first five years since diagnosis (Δ-305 mL/year) that evolves toward a steadier decline after the 10th year (Δ-18 mL/year). Notably, during the total time of follow-up, the patient was frequently admitted to the emergency department complaining of recurrent cough associated with chest infections that were treated with broad-spectrum antibiotics. The general PAM management consisted of daily, high-flow oxygen (up to the current inhaled fraction of 0.40) and prevention of infectious events with vaccines targeting *Streptococcus pneumoniae* or the seasonal flu. She also declined an experimental trial with etidronate, a bisphosphonate that is capable of inhibiting bone mineralization and crystal formation. The drug has been reported to improve the lung function decline along with the diffuse opacification in both lungs. Nowadays, the patient is vitally stable with long-term oxygen therapy and scheduled for follow-up evaluations in the pulmonary clinic.

**Figure 4:**
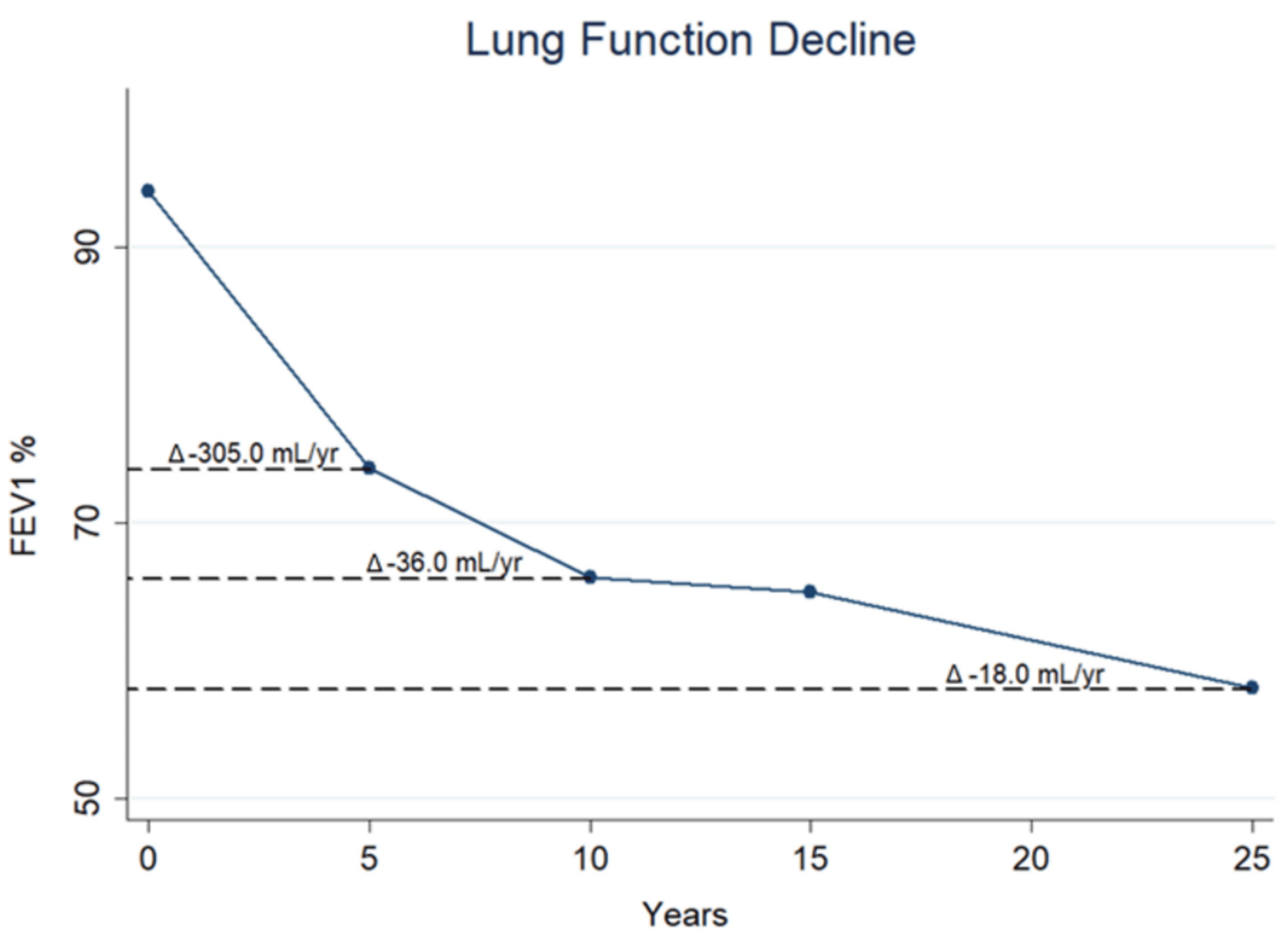
Lung function decline of FEV1 Lung function decline of forced expiratory volume in one second (FEV1) % predicted and relative rate of decline per year (mL/yr) during the long-term follow-up

## Discussion

PAM is a rare, autosomal recessive genetic disorder, and the majority of cases have been reported from Asia and Europe, in particular Turkey, India, and Italy. The prognosis of PAM can be challenging to assess. Adult patients usually show a chronic, progressive decline of lung function leading to respiratory failure and death in mid-life [7]. Yet, cases of a rapidly progressive disease have been reported. A considerable limit in evaluating the lung function decline of patients affected by PAM is given by the severe lack of information about spirometry changes during the disease’s history.

We report a long-term follow-up of a familial case of PAM from our Interstitial Lung Disease Clinic and document the clinical, radiological, and functional progression during the three decades. The hallmark of this genetic condition is the dissociation [8] between clinical symptoms and the severe radiological findings of diffuse centrilobular dense opacities associated with interstitial thickening [9]. An intriguing finding of our case report is the spirometric changes during the follow-up: the patient experienced a rapidly progressive functional decline of FEV1% during the early years of observation that greatly improved from the 10th year up to now. Also, the worsening of the lung function was associated with a deterioration of the clinical status with a persistent dry cough, chest pain, asthenia, and intermittent hemoptysis. Moreover, our series of high-resolution chest CT scans did not exhibit worsening of the diffuse presence of calcium phosphate microliths in both lungs along with the 25-year follow-up.

PAM management was substantially supportive. We administered long-term oxygen therapy for the chronic respiratory failure that occurred in about three years since the diagnosis. Moreover, the patient was admitted to the emergency department of our hospital at least one time per year due to pulmonary infections that were successfully treated using broad-spectrum antibiotics. Previous reports did point out infectious exacerbations as a potential risk factor that may accelerate progression (FV1). Other reports indicated smoke or pollution as potential risk factors for progression (FV2). Nevertheless, the limited decline of the spirometric changes since the 10th year observed in our case suggests that pulmonary infections may not play a role in triggering progression.

Regarding bronchoalveolar lavages and high-dose steroids, as in many previous reports [10], no effectiveness was demonstrated in our case. To the best of our knowledge, the only treatment for end-stage cases is lung transplant, since the possibility of a recurrence has never been described yet. In this specific case, bilateral lung transplant has never been a feasible chance due to the patient’s repeated rejection of the procedure. To date, the patient is scheduled for regular follow-up visits at the institutional pulmonary clinic.

## Conclusions

We report on a PAM familial case with a detailed storyline of lung function decline during three decades of follow-up evaluations. The spirometry changes revealed an unpredictable evolution of the disease with an early stage characterized by a rapidly worsening lung functionality followed by a steadier, though progressive, decline. Dissociation between the clinical and radiological findings was confirmed, and the diffuse, bilateral centrilobular dense opacities were constant during the follow-up. Bronchoalveolar lavage or high-dose steroids had no efficacy, and the option of lung transplant was not accepted by the patient. 

## References

[1] Ferreira Francisco FA, Pereira e Silva JL, Hochhegger B, Zanetti G, Marchiori E (2013). Pulmonary alveolar microlithiasis. State-of-the-art review. Respir Med.

[2] Jönsson ÅL, Simonsen U, Hilberg O, Bendstrup E (2012). Pulmonary alveolar microlithiasis: two case reports and review of the literature. Eur Respir Rev.

[3] Al-Maghrabi H, Mokhtar G, Al-Maghrabi J, Meliti A (2020). Pulmonary alveolar microlithiasis: a report of two unique cases. Respir Med Case Rep.

[4] Castellana G, Castellana G, Gentile M, Castellana R, Resta O (2015). Pulmonary alveolar microlithiasis: review of the 1022 cases reported worldwide. Eur Respir Rev.

[5] Lauta VM (2003). Pulmonary alveolar microlithiasis: an overview of clinical and pathological features together with possible therapies. Respir Med.

[6] Al-Sardar H, Al-Habbo DJ, Al-Hayali RM (2014). Pulmonary alveolar microlithiasis: report of two brothers with the same illness and review of literature. BMJ Case Rep.

[7] Chen CW, Wu FZ (2019). Progressive sandstorm lung in pulmonary alveolar microlithiasis. Ann Thorac Surg.

[8] Cuesta Lujano L, Gutiérrez Domingo Á, Fernández Ollero L (2018). Alveolar microlithiasis and its distinctive clinical and radiological disassociation. Arch Bronconeumol (Engl Ed).

[9] Khaladkar SM, Kondapavuluri SK, Kamal A, Kalra R, Kuber R (2016). Pulmonary alveolar microlithiasis-clinico-radiological dissociation-a case report with radiological review. J Radiol Case Rep.

[10] Mariotta S, Ricci A, Papale M, De Clementi F, Sposato B, Guidi L, Mannino F (2004). Pulmonary alveolar microlithiasis: report on 576 cases published in the literature. Sarcoidosis Vasc Diffuse Lung Dis.

